# Thermogenic Ability of Uncoupling Protein 1 in Beige Adipocytes in Mice

**DOI:** 10.1371/journal.pone.0084229

**Published:** 2013-12-30

**Authors:** Yuko Okamatsu-Ogura, Keigo Fukano, Ayumi Tsubota, Akihiro Uozumi, Akira Terao, Kazuhiro Kimura, Masayuki Saito

**Affiliations:** 1 Department of Biomedical Sciences, Graduate School of Veterinary Medicine, Hokkaido University, Sapporo, Japan; 2 Department of Nutrition, Graduate School of Nursing and Nutrition, Tenshi College, Sapporo, Japan; University of Barcelona, Faculty of Biology, Spain

## Abstract

Chronic adrenergic activation leads to the emergence of beige adipocytes in some depots of white adipose tissue in mice. Despite their morphological similarities to brown adipocytes and their expression of uncoupling protein 1 (UCP1), a thermogenic protein exclusively expressed in brown adipocytes, the beige adipocytes have a gene expression pattern distinct from that of brown adipocytes. However, it is unclear whether the thermogenic function of beige adipocytes is different from that of classical brown adipocytes existing in brown adipose tissue. To examine the thermogenic ability of UCP1 expressed in beige and brown adipocytes, the adipocytes were isolated from the fat depots of C57BL/6J mice housed at 24°C (control group) or 10°C (cold-acclimated group) for 3 weeks. Morphological and gene expression analyses revealed that the adipocytes isolated from brown adipose tissue of both the control and cold-acclimated groups consisted mainly of brown adipocytes. These brown adipocytes contained large amounts of UCP1 and increased their oxygen consumption when stimulated with norepinephirine. Adipocytes isolated from the perigonadal white adipose tissues of both groups and the inguinal white adipose tissue of the control group were white adipocytes that showed no increase in oxygen consumption after norepinephrine stimulation. Adipocytes isolated from the inguinal white adipose tissue of the cold-acclimated group were a mixture of white and beige adipocytes, which expressed UCP1 and increased their oxygen consumption in response to norepinephrine. The UCP1 content and thermogenic ability of beige adipocytes estimated on the basis of their abundance in the cell mixture were similar to those of brown adipocytes. These results revealed that the inducible beige adipocytes have potent thermogenic ability comparable to classical brown adipocytes.

## Introduction

There are two types of fat tissue in mammals, white adipose tissue (WAT) and brown adipose tissue (BAT) [1]–[3]. White adipocytes, a major component of WAT, contain large unilocular triglyceride droplets within the cell. Brown adipocytes, found in BAT, contain a large number of small triglyceride droplets (multilocular) and numerous cristae-rich mitochondria. In addition to these morphological differences, their physiological roles are distinct, particularly in terms of energy metabolism. Norepinephrine (NE) released from sympathetic nerve terminals induces lipolysis in both tissues through the ß-adrenergic receptor. Fatty acids liberated from WAT are released into the blood and used in various tissues such as heart and muscle. In contrast, fatty acids liberated within BAT are oxidized within the tissue itself as a major energy source for thermogenesis. BAT thermogenesis is principally dependent on the activation of uncoupling protein 1 (UCP1), a mitochondrial protein specific to brown adipocytes. UCP1 uncouples mitochondorial oxidative phosphorylation and thereby dissipates the energy from fatty acids as heat. BAT thermogenesis is important for the maintenance of body temperature, particularly in rodents and human newborns [4] as well as human adults [5]–[7]. When mammals are exposed to a cold environment, UCP1 thermogenesis is activated via the sympathetic nerve–NE pathway. Prolonged activation of this pathway induces BAT hyperplasia associated with the elevated UCP1 level [1], [4], [8].

Although WAT and BAT are quite different in morphology and function, the boundary between these tissues is obscure [9]. Chronic adrenergic stimulation, such as cold acclimation or chronic treatment with a ß3-adrenergic receptor agonist, induces UCP1-expressing brown-like adipocytes in specific depots of WAT (WAT browning) [10]–[13]. In addition to UCP1 expression, these brown-like adipocytes have morphological features in common with brown adipocytes, such as multilocular lipid droplets and high mitocondrial content. However, they are considered a different type of adipocyte, designated “beige/brite adipocyte”, because they are derived from a distinct origin and have different gene expression profiles than the classical brown adipocytes [14], [15]. For example, Seal et al. revealed that brown adipocytes are derived from Myf5+ myoblast precursor cells, while beige and white adipocytes arise from cells of the non-Myf5 lineage [16]. Wu et al. identified several genes highly expressed in beige adipocytes but not in brown adipocytes, such as Tbx1, Eva1, and CD137 [17]. Sharp et al. also reported beige adipocyte-selective genes including Cited1 [18]. In addition, human adult BAT has been suggested to resemble beige adipocytes in certain aspects of its gene expression profile [18], [19].

Because a large number of knockout or transgenic mouse models characterized by an emergence of beige adipocytes in WAT are resistant to diet-induced or genetic obesity [20]–[25], it has been assumed that the induction of WAT browning has a significant impact on whole-body energy expenditure. Consistent with these *in vivo* studies, some *in vitro* studies have demonstrated that beige adipocytes differentiated from mouse or human preadipocytes are thermogenically active in response to adrenergic stimulation [26]–[28]. In addition, we reported previously that induction of UCP1 expression in WAT enhances the anorexigenic effect of leptin, an adipokine that reduces food intake and increases energy expenditure [29]. These results suggest that WAT browning is important for resistance to obesity. On the other hand, Nedergaard et al. insisted on a minor role for beige adipocytes in whole-body energy expenditure [30] because the UCP1 expression level in browning WAT is very low compared with that in BAT. This controversy over the significance of beige adipocytes in whole-body energy expenditure arose because, at present, there is no comparative study of the functional differences between inducible beige adipocytes and classical brown adipocytes. Thus, in this study, to evaluate the thermogenic ability of isolated beige adipocytes from the WAT of cold-acclimated mice, we analyzed their UCP1 content and oxygen consumption in comparison with those in brown and white adipocytes.

## Materials and Methods

### Animals and Tissue Sampling

Experimental procedures and animal care were performed in accordance with the Guidelines of Animal Care and Use from Hokkaido University and were approved by the University Committee for the Care and Use of Laboratory Animals. Male 10-week-old C57BL/6J mice were housed in plastic cages placed in an air-conditioned room at 24°C or a cold room at 10°C for 3 weeks. The mice were killed by cervical dislocation, and interscapular BAT, inguinal WAT (I-WAT), and perigonadal WAT (G-WAT) were quickly removed and transferred into liquid nitrogen for western blot analysis or 10% phosphate-buffered formalin for histological examination, or used immediately for the isolation of adipocytes.

### Isolation of Adipocytes

Isolated adipocytes were prepared as reported previously [31] with slight modifications. In brief, tissue fragments were incubated in Krebs-Ringer-HEPES buffer (KRBH) containing 1% fatty acid-free bovine serum albumin (BSA; Sigma Chemical, St. Louis, MO), 2.5 mM glucose and 1 mg/ml collagenase (Wako Pure Chemical Industries, Osaka, Japan) at 37°C for 1 h with shaking at 90 cycles/min. The suspension was filtered through a 200-µm nylon filter and centrifuged at room temperature at 50 *g* for 2 min. The floating cells were washed 3 times with KRBH buffer to eliminate collagenase, brought to a suitable dilution in KRBH buffer containing 4% BSA and 2.7 mM glucose, and kept at room temperature for 1 h before use. The adipocytes were used for the measurement of oxygen consumption and the extraction of total RNA and protein.

### Oxygen Consumption Measurement

The oxygen consumption of the isolated adipocytes was measured polarographically using a Clark-style oxygen electrode in a water-jacketed Perspex chamber at 37°C. The isolated adipocytes were incubated in the chamber in 1 ml KRBH buffer containing 4% BSA and 2.7 mM glucose. Oxygen concentration in the chamber was monitored continuously for 5 min before and 10 min after the addition of 1 µM NE. NE concentration necessary to obtain the maximal effect in brown adipocytes was determined as described previously [32]. Oxygen consumption rates were calculated using computer software (782 System; Strathkelvin Instruments, Glasgow, Scotland). The basal oxygen consumption rate was calculated as the mean of the oxygen consumption rates measured for 5 min before NE stimulation. The NE-induced oxygen consumption rate was calculated by subtracting the basal oxygen consumption rate from the mean of oxygen consumption measured for 10 min after NE stimulation.

### mRNA Analysis

Total RNA was extracted from the isolated adipocytes using the RNAiso reagent (Takara Bio, Shiga, Japan) according to the manufacturer’s protocol. Total RNA (1 µg) was reverse transcribed using a 15-mer oligo(dT) adaptor primer and M-MLV reverse transcriptase (Life Technologies Inc., Carlsbad, CA). Real-time PCR was performed on a fluorescence thermal cycler (LightCycler system; Roche Diagnostics, Mannheim, Germany) using QuantiTect SYBR Green PCR kits (Qiagen Inc., Carlsbad, CA). Absolute expression levels were determined using a standard curve method, with respective cDNA fragments as standards. The levels are reported relative to ß-actin expression. The primers used in this study are listed in Table S1.

### Protein Analysis

Tissue specimens were homogenized in Tris-EDTA buffer (10 mM Tris and 1 mM EDTA, pH 7.4). The isolated adipocytes were washed 3 times with PBS to eliminate BSA, suspended in Tris-EDTA buffer, and frozen at −80°C. Frozen cells were thawed on ice and vortexed briefly. After repeated freeze–thaw steps to complete cell breakage, the sample was centrifuged at 800 *g* for 10 min at 4°C, and the fat-free supernatant was then collected. The resultant pellet was resuspended in Tris-EDTA buffer, incubated on ice for 10 min, and centrifuged at 800 *g* for 10 min. The fat-free supernatant was collected and added to the first portion, and this mixture was centrifuged at 15,000 g for 20 min at 4°C. The final pellet was washed 2 times, suspended in Tris-EDTA buffer, and used for protein concentration measurement and western blot analysis. For western blot analysis, proteins were separated by SDS-PAGE and transferred to a polyvinylidine fluoride membrane (Immobilon; Millipore, Tokyo, Japan). After blocking the membrane with 5% skim milk (Morinaga Milk Industry Co., Tokyo, Japan), it was incubated with a primary anti-rat UCP1 antibody, a kind gift from Dr. Teruo Kawada, or with bovine cytochrome oxidase complex 4 (COX4; Molecular Probes, Eugene, OR) for 1 h. The bound antibody was visualized using horseradish peroxidase-linked goat anti-rabbit immunoglobulin (Zymed Laboratories, San Francisco, CA) for the detection of UCP1 or horseradish peroxidase-linked goat anti-mouse immunoglobulin (Jackson Immunoresearch Laboratories, West Grove, PA) for the detection of COX4 and an enhanced chemiluminescence system (Millipore).

### Histology

Tissue specimens were fixed in 10% formalin, embedded in paraffin, cut into 4-µm-thick sections, and stained with hematoxylin and eosin. Some sections were incubated in 0.3% hydrogen peroxide in methanol to inhibit endogenous peroxidase activity and then with 10% normal goat serum, rabbit antiserum against rat UCP1, goat anti-rabbit IgG (Nichirei, Tokyo, Japan), and finally with the avidin–biotin–peroxidase complex (Nichirei) according to the conventional avidin–biotin complex method.

### Data Analysis

Values are expressed as means ± SE. Statistical analysis was performed using Student’s t-test and ANOVA followed by the Tukey–Kramer post hoc test. Regression analysis and analysis of covariance were also perfomed. All these statistical analyses were performed using StatView statistical software.

## Results

### Effect of Cold Acclimation on Fat Tissues

**Figure 1:**
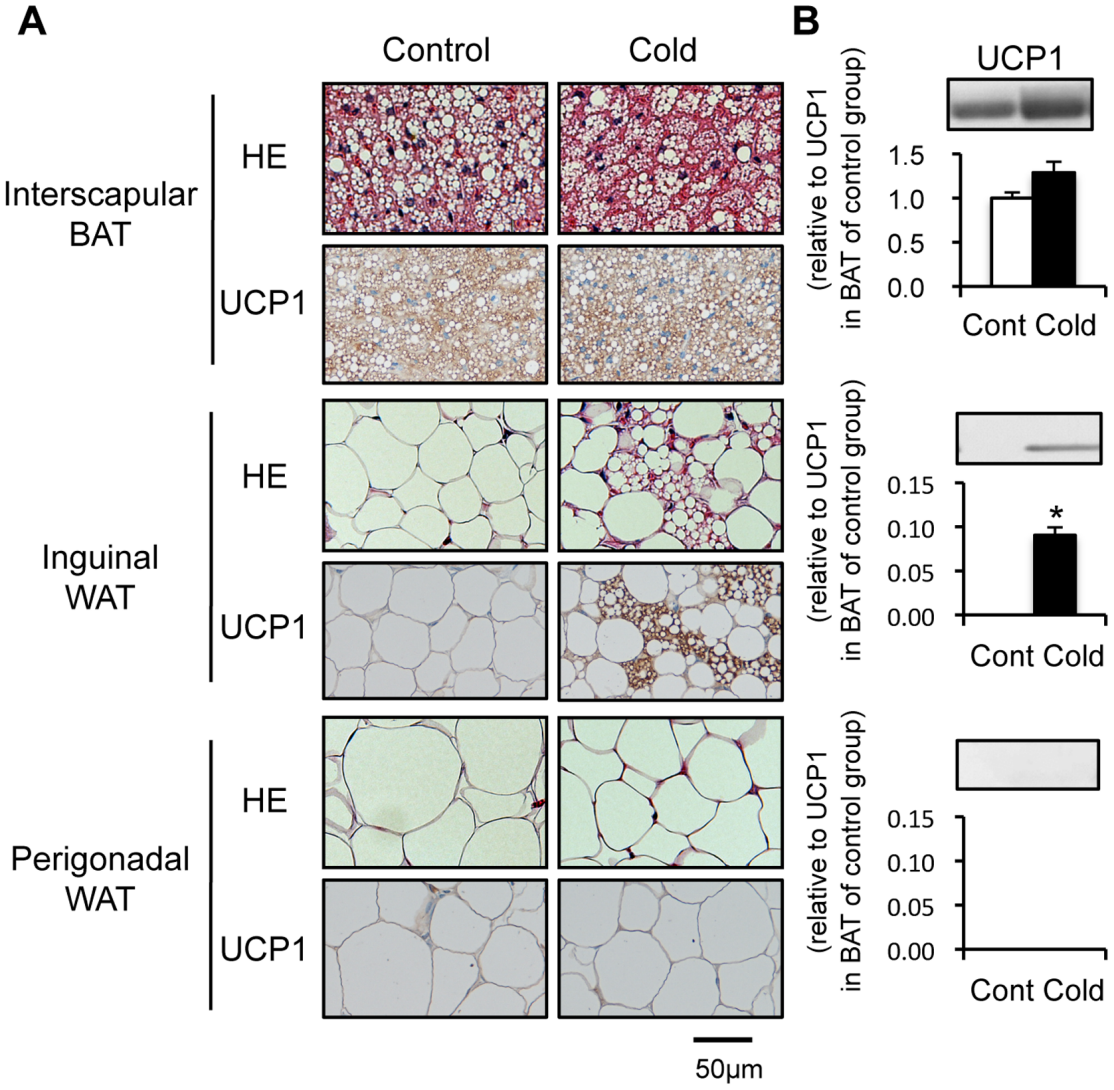
Effect of cold acclimation on cell morphology and uncoupling protein 1 expression in adipose tissues. Mice were housed in a cold environment (10°C; cold-acclimated mice) or a control environment (24°C; control mice) for 3 weeks. (A) Sections of interscapular brown adipose tissue (BAT), inguinal white adipose tissue (WAT) (I-WAT), and perigonadal WAT (G-WAT) were stained with hematoxylin and eosin (HE) or immunostained using antibody against uncoupling protein 1 (UCP1). (B) UCP1 protein expression was analyzed by western blotting. To detect UCP1, 2.5 µg (BAT) or 20 µg (I-WAT and G-WAT) of protein was used. Values are means ± SE for 4 mice. *p<0.05, versus the control group.

As shown in Figure 1A, BAT of C57BL/6 mice housed at 24°C was mainly composed of brown adipocytes with multilocular lipid droplets. I-WAT and G-WAT were composed of unilocular cells filled with a single large lipid droplet, typical of white adipocytes. To induce beige adipocytes in WAT, mice were cold-acclimated at 10°C for 3 weeks. Cold acclimation produced an apparent change in appearance in I-WAT, without notable changes in BAT or G-WAT. In I-WAT of cold-acclimated mice, brown-like adipocytes with multilocular lipid droplets were found surrounded by white adipocytes. Immunostaining showed UCP1 expression in the brown-like adipocytes from I-WAT as well as in the classical brown adipocytes from BAT, while no staining was observed in the unilocular adipocytes from I-WAT or G-WAT.

UCP1 induction in I-WAT was also confirmed by western blotting (Figure 1B). In the control mice, UCP1 was expressed abundantly in BAT but undetectable in I-WAT and G-WAT. Cold acclimation slightly increased UCP1 expression in BAT, but the change was statistically insignificant. In contrast, cold acclimation induced UCP1 expression remarkably in I-WAT, although the protein level was as low as 5–8% of that in BAT of the control mice. Consistent with previous reports showing site-specific induction of UCP1 [33], no UCP1 expression was observed in G-WAT of either the control or cold-acclimated groups. These results confirmed the induction of beige adipocytes in I-WAT after cold acclimation at 10°C for 3 weeks.

### Characteristics of the Isolated Adipocytes from the Three Fat Depots

**Figure 2:**
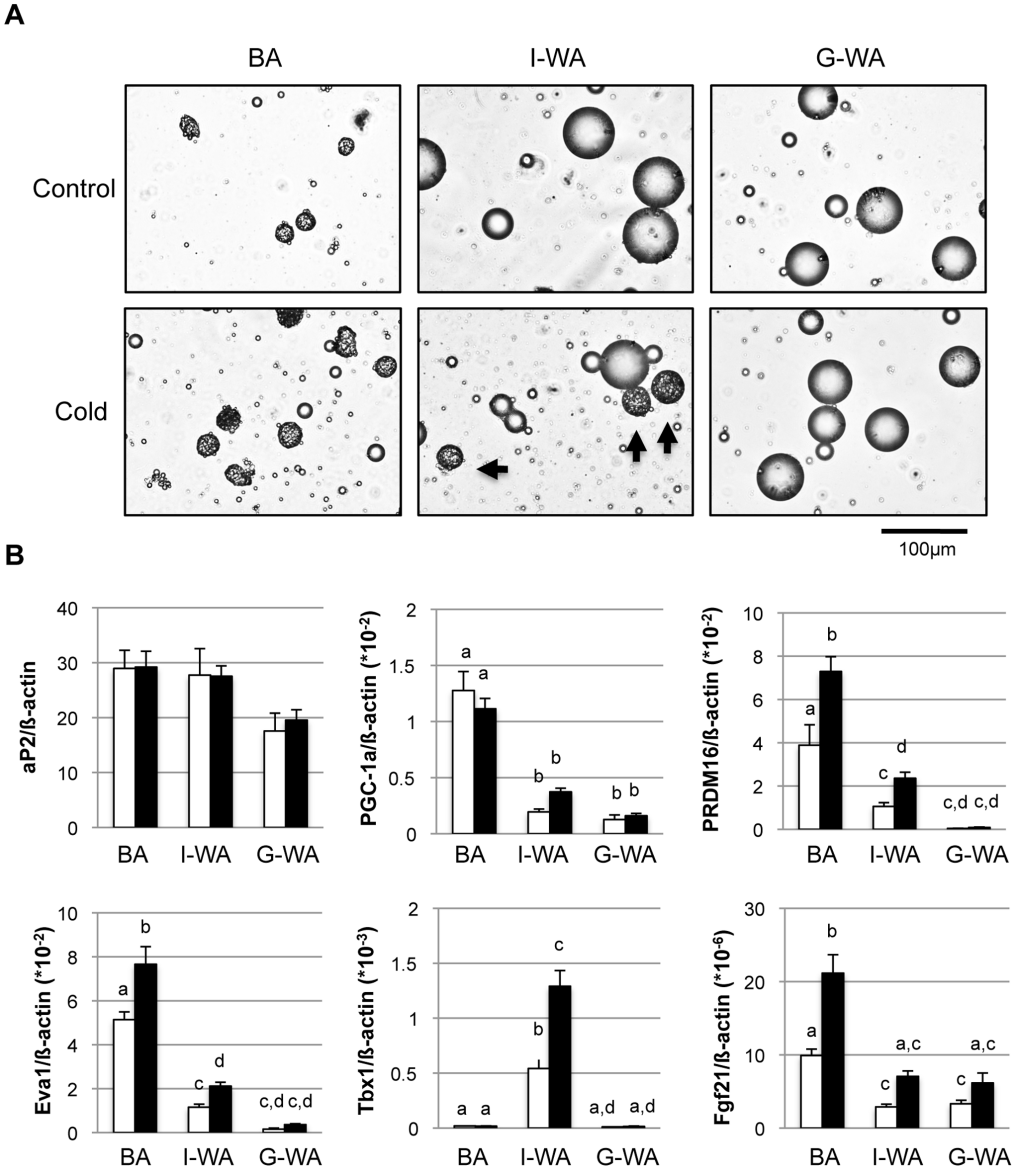
Characteristics of adipocytes isolated from different fat depots of the control and cold-acclimated mice. Adipocytes were isolated from interscapular brown adipose tissue (BA), inguinal (I-WA) and perigonadal white adipose tissue (G-WA) of the control and cold-acclimated mice. (A) Typical photomicrographs of adipocytes isolated form each depot of the control or cold-acclimated mice. Arrows indicate multilocular beige adipocytes. (B) Expression of several genes in the isolated adipocytes was measured by quantitative real-time PCR. Expression levels normalized to ß-actin expression are shown. White and black columns indicate the control and cold-acclimated groups, respectively. Values are means ± SE for 9 mice. Different letters indicate significant differences between the groups (*p*<0.05).

Adipocytes were isolated from BAT, I-WAT, and G-WAT of the control and cold-acclimated mice by collagenase digestion. First, we studied the morphology of the isolated adipocytes (Figure 2A). The adipocytes isolated from BAT (BA) and G-WAT (G-WA) were mainly composed of brown and white adipocytes, respectively, either in the control or cold-acclimated group. Although the adipocytes isolated from I-WAT (I-WA) were composed of white adipocytes in the control group, multilocular adipocytes were found along with the white adipocytes in the cold-acclimated group, indicating that they are the mixture of white and beige adipocytes. The relative abundance of beige adipocytes in the mixture varied 7 to 27%, and its average was 16.5±2.1%. These beige adipocytes tended to be larger in size than the classical brown adipocytes isolated from BAT.

To assess the characteristics of the adipocytes isolated from each depot, we analyzed the expression of several marker genes (Figure 2B). The mature adipocyte marker aP2 was expressed at the same level in the adipocytes isolated from all depots of both the control and cold-acclimated mice, confirming the proper isolation of adipocytes. The expression of PGC-1α, a transcriptional co-factor involved in UCP1 expression and mitochondriagenesis, was high in BA but low in I- and G-WA and did not change after cold acclimation. Brown adipocyte-enriched PRDM16 [34] and Eva1 were expressed at a high level in BA, a relatively low level in I-WA, and an almost undetectable level in G-WA. Cold acclimation significantly increased the expression of both genes in BA and I-WA, but not in G-WA. Beige adipocyte-enriched Tbx1 was expressed only in I-WA and was increased 2.4-fold by cold acclimation. Consistent with a previous report [35], [36], fgf21 was highly expressed in BA compared with I- and G-WA, and cold acclimation increased its expression in all the three depots.

**Figure 3:**
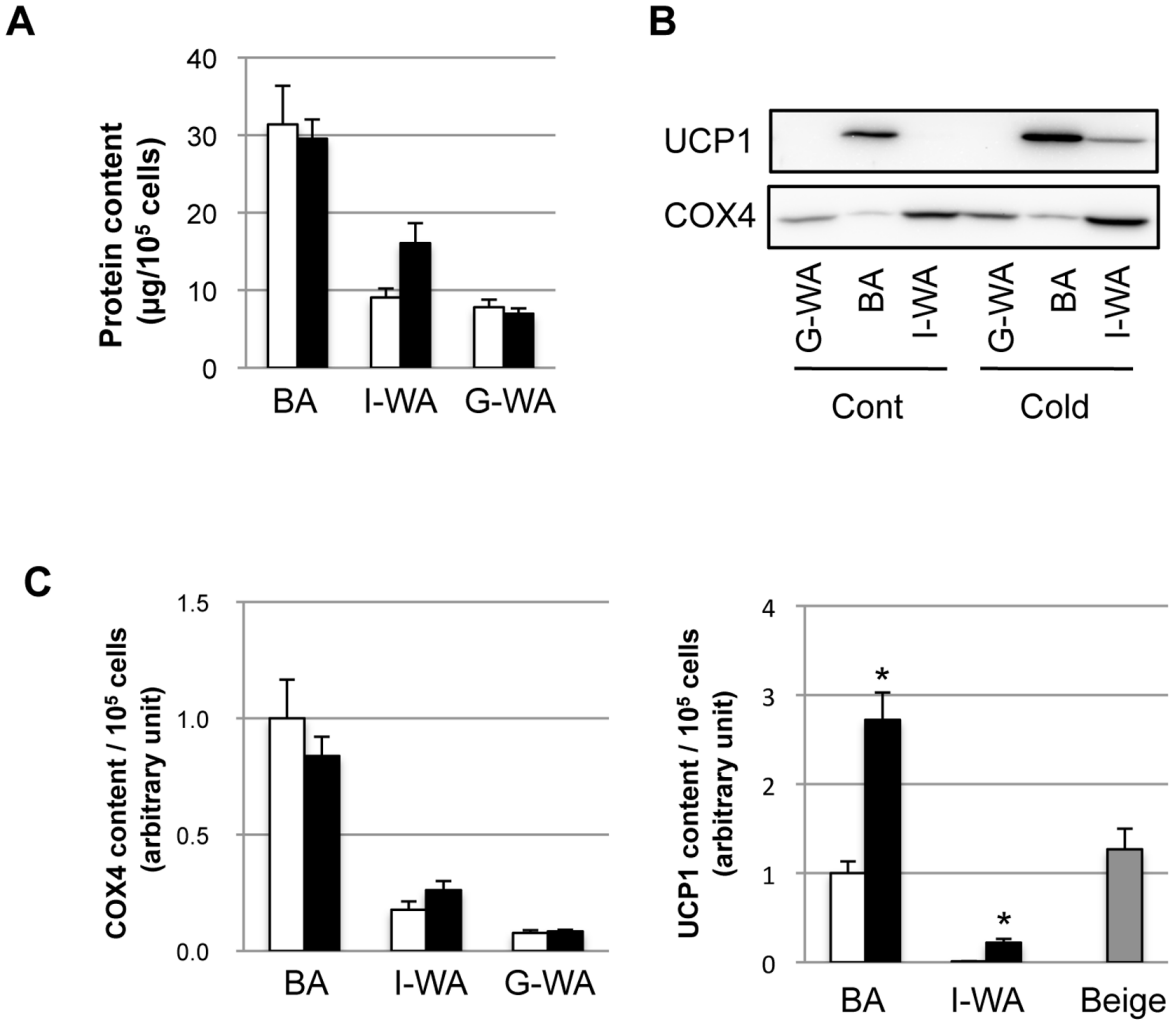
Effect of cold acclimation on the protein content in isolated adipocytes. Protein was extracted from isolated adipocytes from interscapular brown adipose tissue (BA), inguinal (I-WA) and perigonadal white adipose tissue (G-WA) of the control and cold-acclimated mice. (A) Protein content was measured and expressed as the amount of protein from 10^5^ cells. (B) Uncoupling protein 1 (UCP1) and cytochrome oxidase complex 4 (COX4) were detected by western blotting. A typical detection pattern is shown. Protein from BA (1 µg) and WA (10 µg) was loaded on the SDS-PAGE gel. (C) COX4 and UCP1 content per 10^5^ cells were calculated from the protein content (A) and the band density obtained by western blotting (B). White and black columns indicate the control and cold-acclimated groups, respectively. Gray column shows UCP1 content in beige adipocytes estimated on the basis of their abundance in I-WA of the cold-acclimated group. Values are means ± SE for 9 mice. *p<0.05, versus the same depot of the control mice.

Total protein content was higher in BA than in I- and G-WA of the control group (Figure 3A). Cold acclimation had no effect on the protein content in BA or G-WA, but it increased the protein content in I-WA by 1.8-fold. UCP1 and COX4 were detected by western blotting (Figure 3B), and these protein contents were compared in Figure 3C. The COX4 content was not different between the control and cold-acclimated groups in all depots (Figure 3C). The UCP1 content was increased by cold acclimation 2.8- and 20.6-fold in BA and I-WA, respectively. The UCP1 content in I-WA of the cold-acclimated group was as low as 21.9% and 8.1% of that in BA of the control and cold-acclimated groups, respectively. However, as the relative abundance of beige adipocytes in I-WA of the cold-acclimated group was around 17%, their estimated UCP1 content was almost the same as that in BA of the control group, based on the assumption that UCP1 was expressed only in beige adipocytes in I-WA.

### Oxygen Consumption in Adipocytes Isolated from the Three Fat Depots

**Figure 4:**
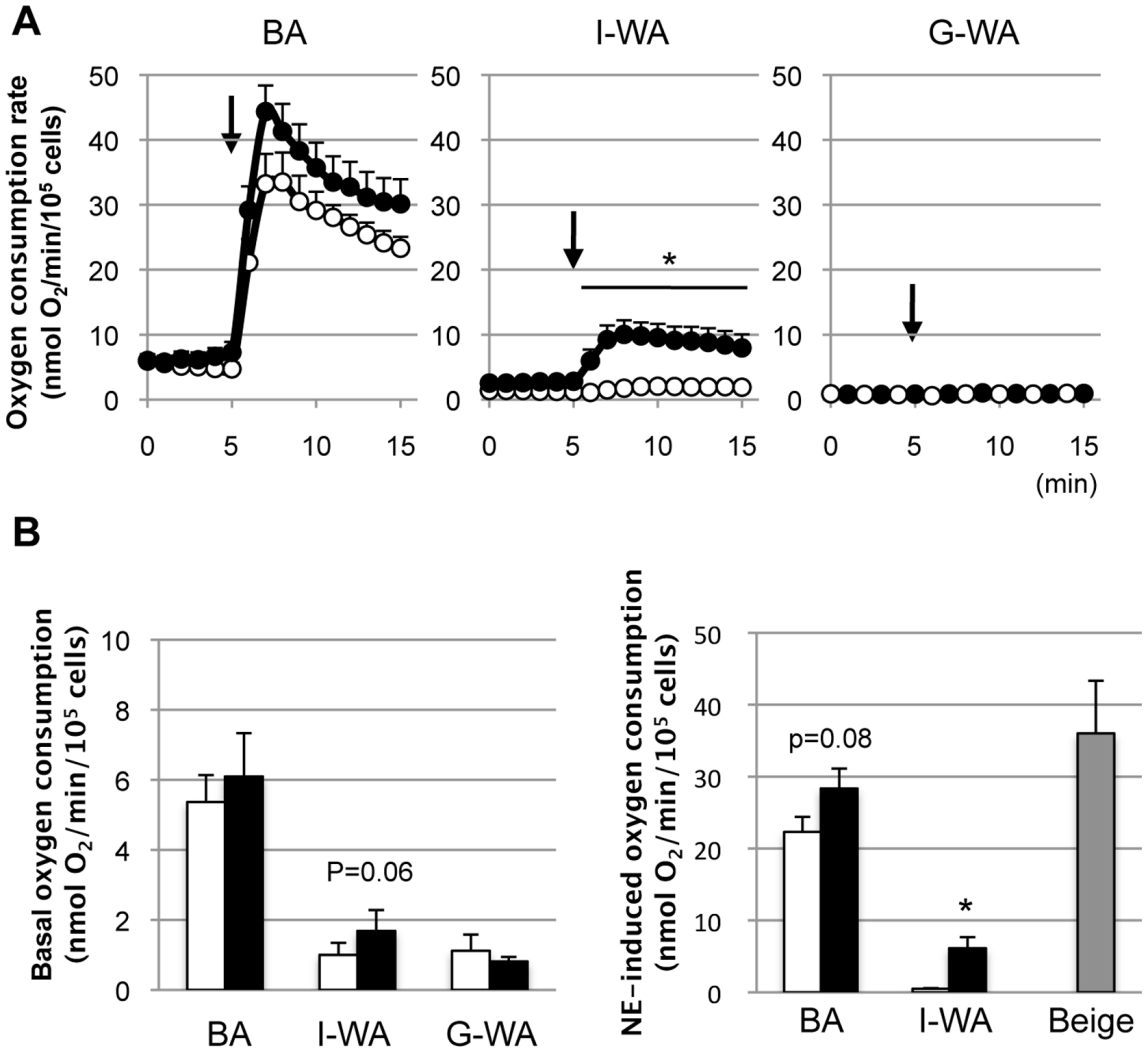
Effect of norepinephrine on the oxygen consumption rate of adipocytes isolated from different fat depots. (A) Oxygen consumption was measured using adipocytes isolated from interscapular brown adipose tissue (BA), inguinal (I-WA) and perigonadal white adipose tissue (G-WA) of the control and cold-acclimated mice. At the arrow (5 min), 1 µM norepinephrine (NE) was added. (B) Basal oxygen consumption rate was calculated and expressed as a mean oxygen consumption rate for 5 min before NE stimulation. NE-induced oxygen consumption rate was calculated as a mean oxygen consumption rate for 10 min after NE stimulation subtracted by basal oxygen consumption rate. White and black columns indicate the control and cold-acclimated groups, respectively. Gray column shows NE-induced oxygen consumption rate of beige adipocytes estimated on the basis of their abundance in I-WA of the cold-acclimated group. Values are means ± SE for 9 mice. *p<0.05, versus the control group of the same tissue.

The basal oxygen consumption rate of BA from the control mice was 5.2±0.8 nmol O_2_/min/10^5^ cells. It increased markedly to 33.5±7.8 nmol O_2_/min/10^5^ cells at 3 min after the addition of NE and decreased gradually thereafter (Figure 4A). The rate remained unchanged after the addition of PBS (data not shown). BA from the cold-acclimated mice showed a slightly higher response to NE, but the difference was statistically insignificant at any point in time. No difference was observed in the basal oxygen consumption rate between the control and cold-acclimated groups. The NE-induced oxygen consumption rate was slightly, but insignificantly, higher in the cold-acclimated group (28.3±2.8 nmol O_2_/min/10^5^ cells) than in the control group (22.3±2.1 nmol O_2_/min/10^5^ cells) (Figure 4B).

The oxygen consumption rate of I-WA from the control group was low at approximately 1.3±0.3 nmol O_2_/min/10^5^ cells and did not change after NE stimulation. The basal oxygen consumption rate of I-WA from the cold-acclimated group (2.7±0.6 nmol O_2_/min/10^5^ cells) was slightly higher than that from the control group, but the difference was not statistically significant (*P* = 0.06). In I-WA of the cold-acclimated group, NE stimulation rapidly increased the oxygen consumption rate to 10.0±2.2 nmol O_2_/min/10^5^ cells in 3 min, and this rate was sustained at that level thereafter (Figure 4A). The NE-induced oxygen consumption rate in the cold-acclimated group was 6.1±1.5 nmol O_2_/min/10^5^ cells, which was approximately 27.5% of that in BA from the control group (Figure 4B). Estimated NE-induced oxygen consumption rate of beige adipocytes was 36.0±7.3 nmol O_2_/min/10^5^ cells, being 1.6-fold higher than that in BA, but not statistically significant. G-WA showed a very low basal oxygen consumption rate (0.8±0.1 nmol O_2_/min/10^5^ cells in both groups), and no response to NE stimulation was observed.

### Relationship of the Oxygen Consumption Rate and Protein Content

**Figure 5:**
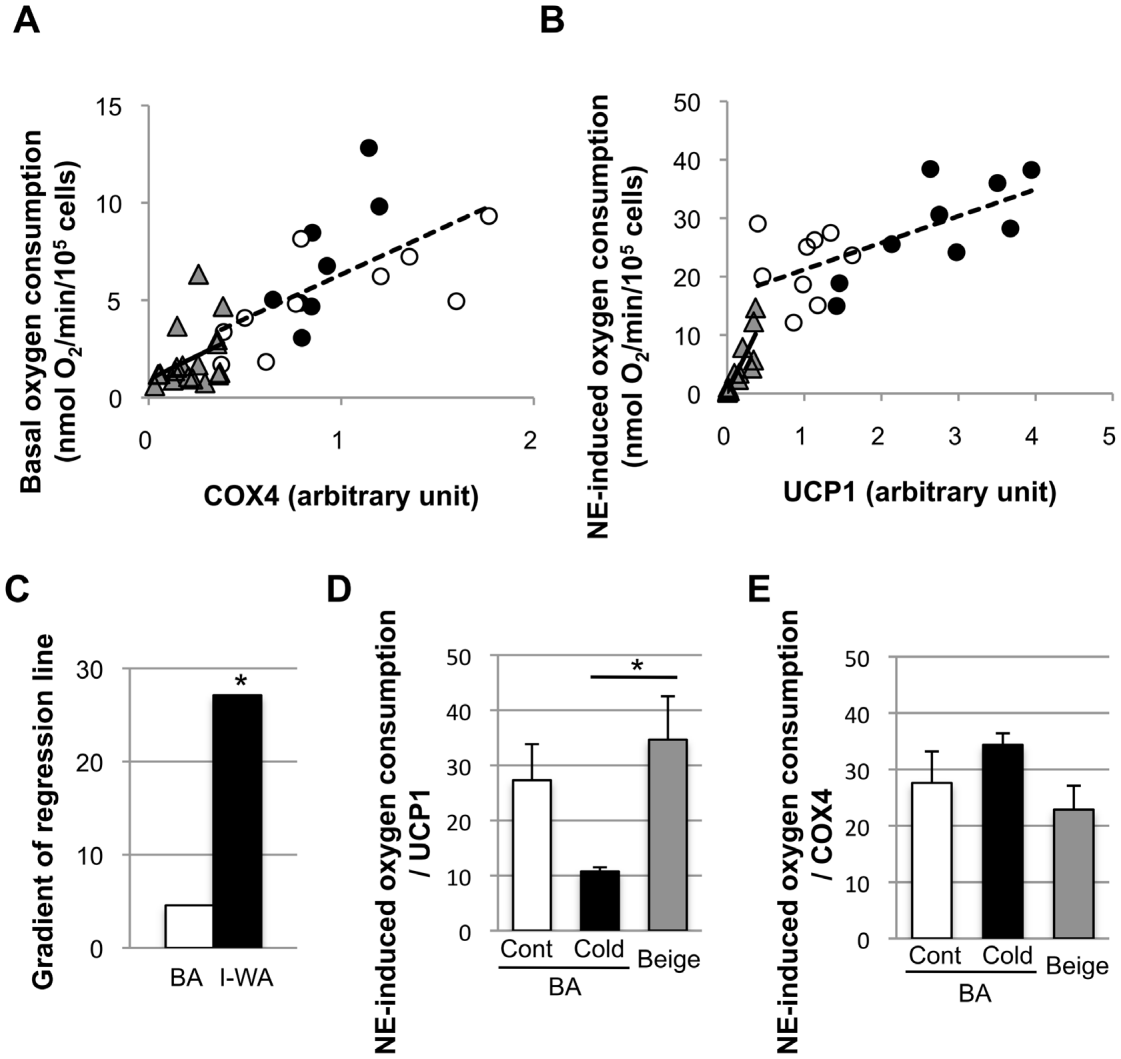
Relationship between the oxygen consumption rate and protein content. The correlations between the basal oxygen consumption rate and cytochrome oxidase complex 4 (COX4) content (A) and between the norepinephrine (NE)-induced oxygen consumption rate and uncoupling protein 1 (UCP1) content (B) in adipocytes isolated from interscapular brown adipose tissue (BA) of the control group (○), of the cold-acclimated group (•), or inguinal white adipose tissue (I-WA: ▴) are shown. Statistical data are shown in Table S2. (C) The gradient of regression lines in (B). The difference between BA and I-WA was compared by ANCOVA. *P<0.05, versus BA. (D) The ability of UCP1 to enhance oxygen consumption by NE stimulation was calculated by dividing the NE-induced oxygen consumption rate by the UCP1 content. (E) NE-induced oxygen consumption per COX4 was calculated. Values are means ± SE for 18 samples. *p<0.05, versus BA.

To assess the possibility that the high content of COX4, an index of mitochondrial content, in BA is related to their high basal oxygen consumption rates, the relationship between the basal oxygen consumption rate and COX4 content was assessed. As shown in Figure 5A, the basal oxygen consumption rate showed a positive correlation with the COX4 content in BA (R = 0.60, *p*<0.01), and the gradient of the regression line was 4.54. In I-WA, the correlation was low and insignificant (R = 0.38, *p* = 0.13), and the gradient of regression line was 4.82.

It has been well accepted that NE-induced oxygen consumption is completely dependent on UCP1 [37], [38]. Therefore, we assessed the correlation between the NE-induced oxygen consumption rate and UCP1 content (Figure 5B). As expected, the NE-induced oxygen consumption rate was positively correlated to the UCP1 content in BA (R = 0.66, *P*<0.01) and I-WA (R = 0.88, *P*<0.01) (Table S2). The gradient of the regression line was 5.9-fold greater in I-WA (27.1) than in BA (4.57), and ANCOVA revealed that the difference was statistically significant (Figure 5C). The thermogenic ability of UCP1 calculated simply by dividing the NE-induced oxygen consumption rate by the UCP1 content was 34.7±7.9 nmol O_2_/min/UCP1 in I-WA of the cold-acclimated group, which was comparable to BA of the control group (27.3±6.5 nmol O_2_/min/UCP1), but higher than BA of the cold-acclimated group (10.8±0.7 nmol O_2_/min/UCP1) (Figure 5D). NE-induced oxygen consumption rate per COX4 was not different among the three groups (Figure 5E).

## Discussion

It is well established that beige adipocytes are induced in WAT after chronic ß-adrenergic activation [10]–[13]. Despite recent advances in the molecular understanding of their origins or progenitor cells [39], [40], of the differentiation mechanisms for brown and beige adipcoytes [26], [34], and of inducers of WAT browning [36], [40]–[42], it has been unclear whether the function of the beige adipocytes is different from, or basically the same as, that of classical brown adipocytes. We have demonstrated that the beige adipocytes isolated from I-WAT of the cold-acclimated mice express comparable amount of UCP1 to classical brown adipocytes, and have potent thermogenic ability.

To obtain three types of adipocytes (brown, beige, and white), adipocytes were isolated from BAT, I-WAT, and G-WAT of the control and cold-acclimated mice. In both the control and cold-acclimated groups, BA consisted primarily of brown adipocytes that have multilocular oil droplets within the cell, have high UCP1 and mitochondria (COX4) content, and express brown adipocyte-enriched genes such as PGC-1a, PRDM16, and Eva1. G-WA from either the control or cold-acclimated group were white adipocytes with unilocular oil droplets. I-WA were white adipocytes in the control group, but a mixture of beige and white adipocytes in the cold-acclimated group, and the relative abundance of beige adipocytes in the mixture was around 17% on average. Although it was difficult to distinguish the beige adipocytes from the classical brown adipocytes by their cell morphology, they expressed the beige adipocyte-enriched gene Tbx1, indicating that beige adipocytes had a different gene expression profile from classical brown adipocytes.

The basal oxygen consumption rate without NE stimulation was higher in BA than in I-WA, being proportional to the COX4 content in almost the same manner. This suggests that the basal oxygen consumption rate is largely dependent on the number of mitochondria. When cells were stimulated with NE, oxygen consumption was greatly increased in both BA and I-WA from the cold-acclimated group expressing UCP1, but not in I-WA from the control group or in G-WA expressing no UCP1. These results, being consistent with the reports that brown adipocytes isolated from UCP1-KO mice show no response to NE in oxygen consumption [37], [38], confirm that the NE-induced increment in oxygen consumption is fully dependent on UCP1. In our study, beige adipocytes expressed comparable amount of UCP1 to control classical BA and showed similar, rather higher NE-induced oxygen consumption. These results indicate that beige adipocytes have comparable thermogenic ability to classical brown adipocytes.

Interestingly, although the NE-induced oxygen consumption rate was positively correlated with the UCP1 content, the regression lines for BA and I-WA were significantly different, showing a 5.9-fold higher gradient in I-WA than in BA. Estimation of NE-induced oxygen consumption per UCP1 revealed that thermogenic ability of UCP1 was decreased by cold acclimation in BA. Although the reason for such decrease is unclear, there are several possibilities. One is that there may be some limitation to the maximal thermogenic ability of UCP1. It has been reported that UCP1 overexpression, in excess of the capacity of the mitochodrial respiratory machinery, is toxic to cells. For example, while transgenic mice that over-express UCP1 from the adipocyte-specific aP2 promoter (aP2-UCP1 mice) are resistant to high fat diet-induced obesity because of the ectopic expression of UCP-1 in WAT, the excessive expression of UCP1 from the transgene also induced BAT atrophy [43]. Muscle-specific UCP1 expression in mice resulted in mitochondrial myopathy, with structurally abnormal mitochondria found only in the transgenic line of mice with higher expression levels of UCP1 [44]–[46]. Thus, it seems likely that UCP1 activity is restricted through some mechanism, or mechanisms, in BA of the cold-acclimated group to avoid cytotoxicity. Alternatively, it would be also possible that the thermogenic capacity of UCP1 is limited by the mitochondrial oxidative capacity. On the basis of the characterization of aP2-UCP1 mice, Baumruk et al. reproted that full uncoupling activity of ectopic UCP1 in WAT was achieved by approximately 15-fold lesser UCP1 expression than that in BAT; the ratio of the UCP1 molecule to the mitochondrial respiratory chain being approximately 1, whereas the ratio in BAT was between 5 to 11 [47], [48]. Lin and Klingenberg also suggested that the protonophoric activity of UCP1 exceeds the proton-pumping activity of the respiratory chain in the native mitochondrial membrane several-fold [49]. Collectively, excessive amounts of UCP1 above the capacity of respiratory chain may fail to induce full uncoupling activity of each UCP1 molecule. Consistent with this idea, in our present study, cold acclimation increased UCP1 content in BA 2.7-fold, whereas COX4 level did not change, indicating that UCP1 content per mitochondrion was considerably increased in the cold-acclimated group. This is also suppoted by our result that NE-induced oxygen consmption per mitochondrion (COX4) and per cell did not change with cold-acclimation. Another explanation is that the sensitivity to NE was changed by cold acclimation. It has been reported that continuous adrenergic stimulation resulted in the reduction of the sensitivity to NE in brown adipocytes [1], [50], [51]. Consistant with this, we also confirmed in preliminary experiments that NE-induced fatty acid release was lower in BA of the cold-acclimated group than the control group (data not shown).

Our present results indicate that UCP1 in beige adipocytes has high thermogenic ability similar to that in classical brown adipocytes. However, considering that the UCP1 expression level in WAT induced by cold acclimation is as low as 10% of that in BAT, it would be possible that the contribution of beige adipocytes to the whole-body energy expenditure is low, as suggested by Nedergaard et al [30]. However, accumulating evidence suggests a potential role for beige adipocytes within WAT in whole-body energy expenditure and protection against obesity. For example, as mentioned above, the aP2-UCP1 transgenic mice are resistant to diet-induced obesity, in spite of BAT atrophy [43]. Besides, the emergence of beige adipocytes in WAT has been reported to be associated with a lean phenotype in several lines of transgenic mice [20]–[25]. Moreover, beige adipocytes within WAT were characteristic of a mouse strain resistant to obesity, such as the A/J strain [52], [53]. It is thus possible that beige adipocytes have other roles to control adiposity besides energy expenditure, for example, through the secretion of some adipokines. Further investigation *in vivo* is required to estimate the contribution of beige adipocytes to whole-body energy expenditure.

In summary, our study demonstrates that UCP1 in beige adipocytes induced within WAT have potent thermogenic ability similar to that in classical brown adipocytes. This is the first report that provides comparative analysis of the function of beige and classical brown adipocytes.

## Supporting Information

Table S1 Primer sequences for the real-time PCR.
(DOCX)

Table S2 Statistical information in the regression analysis of the oxygen consumption rate and protein content in adipocytes.
(DOCX)

## References

[1] Cannon B, Nedergaard J (2004) Brown adipose tissue: function and physiological significance. Physiol Rev 84(1): 277–359. 14715917 10.1152/physrev.00015.2003

[2] Frontini A, Cinti S (2010) Distribution and development of brown adipocytes in the murine and human adipose organ. Cell Metab 11(4): 253–256. 20374956 10.1016/j.cmet.2010.03.004

[3] Cinti S (2012) The adipose organ at a glance. Dis Model Mech 5(5): 588–594. 22915020 10.1242/dmm.009662 PMC3424455

[4] Klingenspor M (2003) Cold-induced recruitment of brown adipose tissue thermogenesis. Exp Physiol 88(1): 141–148. 12525862 10.1113/eph8802508

[5] Saito M, Okamatsu-Ogura Y, Matsushita M, Watanabe K, Yoneshiro T, et al (2009) High incidence of metabolically active brown adipose tissue in healthy adult humans: effects of cold exposure and adiposity. Diabetes 58(7): 1526–1531. 19401428 10.2337/db09-0530 PMC2699872

[6] Enerbäck S (2010) Human brown adipose tissue. Cell Metab 11(4): 248–252. 20374955 10.1016/j.cmet.2010.03.008

[7] Yoneshiro T, Aita S, Matsushita M, Kayahara T, Kameya T, et al. Recruited brown adipose tissue as an antiobesity agent in humans. J Clin Invest 123(8): 3404–3408. 23867622 10.1172/JCI67803 PMC3726164

[8] Seale P, Kajimura S, Spiegelman BM (2009) Transcriptional control of brown adipocyte development and physiological function–of mice and men. Genes Dev 23(7): 788–797. 19339685 10.1101/gad.1779209 PMC2763499

[9] Cinti S (2009) Transdifferentiation properties of adipocytes in the adipose organ. Am J Physiol Endocrinol Metab 297(5): E977–E986. 19458063 10.1152/ajpendo.00183.2009

[10] Cousin B, Cinti S, Morroni M, Raimbault S, Ricquier D, et al (1992) Occurrence of brown adipocytes in rat white adipose tissue: molecular and morphological characterization. J Cell Sci 103 (Pt 4): 931–942. 10.1242/jcs.103.4.931 1362571

[11] Ghorbani M, Himms-Hagen J (1997) Appearance of brown adipocytes in white adipose tissue during CL 316,243-induced reversal of obesity and diabetes in Zucker fa/fa rats. Int J Obes Relat Metab Disord 21(6): 465–475. 9192230 10.1038/sj.ijo.0800432

[12] Inokuma K, Okamatsu-Ogura Y, Omachi A, Matsushita Y, Kimura K, et al (2006) Indispensable role of mitochondrial UCP1 for antiobesity effect of beta3-adrenergic stimulation. Am J Physiol Endocrinol Metab 290(5): E1014–E1021. 16368788 10.1152/ajpendo.00105.2005

[13] Barbatelli G, Murano I, Madsen L, Hao Q, Jimenez M, et al (2010) The emergence of cold-induced brown adipocytes in mouse white fat depots is determined predominantly by white to brown adipocyte transdifferentiation. Am J Physiol Endocrinol Metab 298(6): E1244–E1253. 20354155 10.1152/ajpendo.00600.2009

[14] Kajimura S, Seale P, Spiegelman BM (2010) Transcriptional control of brown fat development. Cell Metab 11(4): 257–262. 20374957 10.1016/j.cmet.2010.03.005 PMC2857670

[15] Petrovic N, Walden TB, Shabalina IG, Timmons JA, Cannon B, et al (2010) Chronic peroxisome proliferator-activated receptor gamma (PPARgamma) activation of epididymally derived white adipocyte cultures reveals a population of thermogenically competent, UCP1-containing adipocytes molecularly distinct from classic brown adipocytes. J Biol Chem 285(10): 7153–7164. 20028987 10.1074/jbc.M109.053942 PMC2844165

[16] Seale P, Kajimura S, Yang W, Chin S, Rohas LM, et al (2007) Transcriptional control of brown fat determination by PRDM16. Cell Metab 6(1): 38–54. 17618855 10.1016/j.cmet.2007.06.001 PMC2564846

[17] Wu J, Boström P, Sparks LM, Ye L, Choi JH, et al (2012) Beige adipocytes are a distinct type of thermogenic fat cell in mouse and human. Cell 150(2): 366–376. 22796012 10.1016/j.cell.2012.05.016 PMC3402601

[18] Sharp LZ, Shinoda K, Ohno H, Scheel DW, Tomoda E, et al (2012) Human BAT possesses molecular signatures that resemble beige/brite cells. PLoS One 7(11): e49452. 23166672 10.1371/journal.pone.0049452 PMC3500293

[19] Lidell ME, Betz MJ, Dahlqvist Leinhard O, Heglind M, Elander L, et al (2013) Evidence for two types of brown adipose tissue in humans. Nat Med 19(5): 631–634. 23603813 10.1038/nm.3017

[20] Kopecky J, Clarke G, Enerbäck S, Spiegelman B, Kozak LP (1995) Expression of the mitochondrial uncoupling protein gene from the aP2 gene promoter prevents genetic obesity. J Clin Invest 96(6): 2914–2923. 8675663 10.1172/JCI118363 PMC186003

[21] Soloveva V, Graves RA, Rasenick MM, Spiegelman BM, Ross SR (1997) Transgenic mice overexpressing the beta 1-adrenergic receptor in adipose tissue are resistant to obesity. Mol Endocrinol 11(1): 27–38. 8994185 10.1210/mend.11.1.9870

[22] Tsukiyama-Kohara K, Poulin F, Kohara M, DeMaria CT, Cheng A, et al (2001) Adipose tissue reduction in mice lacking the translational inhibitor 4E-BP1. Nat Med 7(10): 1128–1132. 11590436 10.1038/nm1001-1128

[23] Leonardsson G, Steel JH, Christian M, Pocock V, Milligan S, et al (2004) Nuclear receptor corepressor RIP140 regulates fat accumulation. Proc Natl Acad Sci U S A 101(22): 8437–8442. 15155905 10.1073/pnas.0401013101 PMC420412

[24] Scimè A, Grenier G, Huh MS, Gillespie MA, Bevilacqua L, et al (2005) Rb and p107 regulate preadipocyte differentiation into white versus brown fat through repression of PGC-1alpha. Cell Metab 2(5): 283–295. 16271529 10.1016/j.cmet.2005.10.002

[25] Auffret J, Viengchareun S, Carré N, Denis RG, Magnan C, et al (2012) Beige differentiation of adipose depots in mice lacking prolactin receptor protects against high-fat-diet-induced obesity. FASEB J 26(9): 3728–3237. 22637534 10.1096/fj.12-204958

[26] Seale P, Conroe HM, Estall J, Kajimura S, Frontini A, et al (2011) Prdm16 determines the thermogenic program of subcutaneous white adipose tissue in mice. J Clin Invest 121(1): 96–105. 21123942 10.1172/JCI44271 PMC3007155

[27] Ohno H, Shinoda K, Spiegelman BM, Kajimura S (2012) PPARγ agonists induce a white-to-brown fat conversion through stabilization of PRDM16 protein. Cell Metab 15(3): 395–404. 22405074 10.1016/j.cmet.2012.01.019 PMC3410936

[28] Lee P, Werner CD, Kebebew E, Celi FS. (2013) Functional thermogenic beige adipogenesis is inducible in human neck fat. Int J Obes (Lond). In press. 10.1038/ijo.2013.82 PMC7034754 23736373

[29] Okamatsu-Ogura Y, Nio-Kobayashi J, Iwanaga T, Terao A, Kimura K, et al (2011) Possible involvement of uncoupling protein 1 in appetite control by leptin. Exp Biol Med (Maywood) 236(11): 1274–1281. 21987829 10.1258/ebm.2011.011143

[30] Nedergaard J, Cannon B (2013) UCP1 mRNA does not produce heat. Biochim Biophys Acta 1831(5): 943–949. 23353596 10.1016/j.bbalip.2013.01.009

[31] Omachi A, Matsushita Y, Kimura K, Saito M (2008) Role of uncoupling protein 1 in the anti-obesity effect of beta3-adrenergic agonist in the dog. Res Vet Sci 85(2): 214–219. 18406437 10.1016/j.rvsc.2007.11.003

[32] Shimada K, Ohno Y, Okamatsu-Ogura Y, Suzuki M, Kamikawa A, Terao A, et al (2012) Neuropeptide Y activates phosphorylation of ERK and STAT3 in stromal vascular cells from brown adipose tissue, but fails to affect thermogenic function of brown adipocytes. Peptides 34(2): 336–342. 22374387 10.1016/j.peptides.2012.02.012

[33] Collins S, Daniel KW, Petro AE, Surwit RS (1997) Strain-specific response to beta 3-adrenergic receptor agonist treatment of diet-induced obesity in mice. Endocrinology. 138(1): 405–413. 10.1210/endo.138.1.4829 8977430

[34] Seale P, Bjork B, Yang W, Kajimura S, Chin S, et al (2008) PRDM16 controls a brown fat/skeletal muscle switch. Nature 454(7207): 961–967. 18719582 10.1038/nature07182 PMC2583329

[35] Hondares E, Iglesias R, Giralt A, Gonzalez FJ, Giralt M, et al (2011) Thermogenic activation induces FGF21 expression and release in brown adipose tissue. J Biol Chem 286(15): 12983–12990. 21317437 10.1074/jbc.M110.215889 PMC3075644

[36] Fisher FM, Kleiner S, Douris N, Fox EC, Mepani RJ, et al (2012) FGF21 regulates PGC-1α and browning of white adipose tissues in adaptive thermogenesis. Genes Dev 26(3): 271–281. 22302939 10.1101/gad.177857.111 PMC3278894

[37] Golozoubova V, Hohtola E, Matthias A, Jacobsson A, Cannon B, et al (2001) Only UCP1 can mediate adaptive nonshivering thermogenesis in the cold. FASEB J 15(11): 2048–2050. 11511509 10.1096/fj.00-0536fje

[38] Nedergaard J, Golozoubova V, Matthias A, Asadi A, Jacobsson A, et al (2001) UCP1: the only protein able to mediate adaptive non-shivering thermogenesis and metabolic inefficiency. Biochim Biophys Acta 1504(1): 82–106. 11239487 10.1016/s0005-2728(00)00247-4

[39] Schulz TJ, Huang TL, Tran TT, Zhang H, Townsend KL, et al (2011) Identification of inducible brown adipocyte progenitors residing in skeletal muscle and white fat. Proc Natl Acad Sci U S A 108(1): 143–148. 21173238 10.1073/pnas.1010929108 PMC3017184

[40] Tran KV, Gealekman O, Frontini A, Zingaretti MC, Morroni M, et al (2012) The vascular endothelium of the adipose tissue gives rise to both white and brown fat cells. Cell Metab 15(2): 222–229. 22326223 10.1016/j.cmet.2012.01.008 PMC3278718

[41] Bordicchia M, Liu D, Amri EZ, Ailhaud G, Dessì-Fulgheri P, et al (2012) Cardiac natriuretic peptides act via p38 MAPK to induce the brown fat thermogenic program in mouse and human adipocytes. J Clin Invest 122(3): 1022–1036. 22307324 10.1172/JCI59701 PMC3287224

[42] Boström P, Wu J, Jedrychowski MP, Korde A, Ye L, et al (2012) Lo JC, Rasbach KA, Boström EA, Choi JH, Long JZ, Kajimura S, Zingaretti MC, Vind BF, Tu H, Cinti S, Højlund K, Gygi SP, Spiegelman BM. A PGC1-α-dependent myokine that drives brown-fat-like development of white fat and thermogenesis. Nature 481(7382): 463–468. 22237023 10.1038/nature10777 PMC3522098

[43] Stefl B, Janovská A, Hodný Z, Rossmeisl M, Horáková M, et al (1998) Brown fat is essential for cold-induced thermogenesis but not for obesity resistance in aP2-Ucp mice. Am J Physiol 274(3 Pt 1): E527–E533. 10.1152/ajpendo.1998.274.3.E527 9530137

[44] Li B, Nolte LA, Ju JS, Han DH, Coleman T, et al (2000) Skeletal muscle respiratory unco upling prevents diet-induced obesity and insulin resistance in mice. Nat Med 6(10): 1115–1120. 11017142 10.1038/80450

[45] Han DH, Nolte LA, Ju JS, Coleman T, Holloszy JO, et al (2004) UCP-mediated energy depletion in skeletal muscle increases glucose transport despite lipid accumulation and mitochondrial dysfunction. Am J Physiol Endocrinol Metab 286(3): E347–E353. 14613927 10.1152/ajpendo.00434.2003

[46] Dupuis L, Gonzalez de Aguilar JL, Echaniz-Laguna A, Eschbach J, Rene F, et al (2009) Muscle mitochondrial uncoupling dismantles neuromuscular junction and triggers distal degeneration of motor neurons. PLoS One 4(4): e5390. 19404401 10.1371/journal.pone.0005390 PMC2671839

[47] Baumruk F, Flachs P, Horáková M, Floryk D, Kopecký J (1999) Transgenic UCP1 in white adipocytes modulates mitochondrial membrane potential. FEBS Lett. 444(2–3): 206–210. 10.1016/s0014-5793(99)00053-8 10050760

[48] Flachs P, Rossmeisl M, Kuda O, Kopecky J (2013) Stimulation of mitochondrial oxidative capacity in white fat independent of UCP1: a key to lean phenotype. Biochim Biophys Acta. 1831(5): 986–1003. 10.1016/j.bbalip.2013.02.003 23454373

[49] Lin CS, Klingenberg M (1982) Characteristics of the isolated purine nucleotide binding protein from brown fat mitochondria. Biochemistry. 21(12): 2950–2956. 10.1021/bi00541a023 7104305

[50] Nedergaard J (1982) Catecholamine sensitivity in brown fat cells from cold-acclimated hamsters and rats. Am J Physiol 242(3): C250–C257. 6278947 10.1152/ajpcell.1982.242.3.C250

[51] Svartengren J, Svoboda P, Cannon B (1982) Desensitisation of beta-adrenergic responsiveness in vivo. Decreased coupling between receptors and adenylate cyclase in isolated brown-fat cells. Eur J Biochem 128(2–3): 481–488. 6295759

[52] Guerra C, Koza RA, Yamashita H, Walsh K, Kozak LP (1998) Emergence of brown adipocytes in white fat in mice is under genetic control. Effects on body weight and adiposity. J Clin Invest 102(2): 412–420. 9664083 10.1172/JCI3155 PMC508900

[53] Xue B, Rim JS, Hogan JC, Coulter AA, Koza RA, et al (2007) Genetic variability affects the development of brown adipocytes in white fat but not in interscapular brown fat. J Lipid Res 48(1): 41–51. 17041251 10.1194/jlr.M600287-JLR200

