# Supplementary material for: Thermogenic Ability of Uncoupling Protein 1 in Beige Adipocytes in Mice
Source: PLoS One. 2013 Dec 30;8(12):e84229. doi: 10.1371/journal.pone.0084229 (PMC3875535; doi:10.1371/journal.pone.0084229)
Supplement: Table S1 — Primer sequences for the real-time PCR. (DOCX) [file pone.0084229.s001.docx]

**Table S1 Primer sequences for the real-time PCR**

| Gene | Forward primer | Reverse primer |
| --- | --- | --- |
| ß-actin | 5′-TCG TTAC CAC AGG CAT TGT GAT-3′ | 5′-TGC TCG AAG TCT AGA GCA AC-3′ |
| aP2 | 5′-AAGACAGCTCCTCCTCGAAGGTT-3′ | 5′-TGACCAAATCCCCATTTACGC-3′ |
| PGC-1a | 5′-GTGTGGAACTCTCTGGAACT-3′ | 5′-GCGTACAACTCAGATTGCTC-3′ |
| Prdm16 | 5′-GACATTCCAATCCCACCAGA-3′ | 5′-CACCTCTGTATCCGTCAGCA-3′ |
| Eva1 | 5′-CCA CTT CTC CTG AGT TTA CAG C-3′ | 5′-GCA TTT TAA CCG AAC ATC TGT CC-3′ |
| Tbx1 | 5′-GGC AGG CAG ACG AAT GTT C-3′ | 5′-TTG TCA TCT ACG GGC ACA AAG-3′ |
| Fgf21 | 5′-CTG CTG GGG GTC TAC CAA G-3′ | 5′-CTG CGC CTA CCA CTG TTC C-3′ |
