# Supplementary material for: Thermogenic Ability of Uncoupling Protein 1 in Beige Adipocytes in Mice
Source: PLoS One. 2013 Dec 30;8(12):e84229. doi: 10.1371/journal.pone.0084229 (PMC3875535; doi:10.1371/journal.pone.0084229)
Supplement: Table S2 — Statistical information in the regression analysis of the oxygen consumption rate and protein content in adipocytes. (DOCX) [file pone.0084229.s002.docx]

**Table S2**

**Statistical information in the regression analysis of the oxygen consumption rate and protein content in adipocytes**

| Adipocyte | n | Correlation coefficient (R) | | Gradient of the regression line | *P* value |
| --- | --- | --- | --- | --- | --- |
| Basal oxygen consumption rate vs. COX4 | | | | | |
| BA | 18 | | 0.599 | 4.54 | 0.0086 |
| I-WA | 18 | | 0.375 | 4.82 | 0.1253 |
| BA + I-WA | 36 | | 0.775 | 5.24 | <0.0001 |
| NE-induced oxygen consumption rate vs. UCP1 | | | | | |
| BA | 18 | | 0.663 | 4.57 | 0.0027 |
| I-WA | 18 | | 0.877 | 27.11 | <0.0001 |
| BA + I-WA | 36 | | 0.856 | 9.16 | <0.0001 |

BA, adipocytes isolated from interscapular brown adipose tissue; I-WA, adipocytes isolated from inguinal whit adipose tissue; NE, norepinephrine; COX4, cytochrome oxidase complex 4; UCP1, uncoupling protein 1
